# Validation of the CALL score as a mortality prediction tool in a cohort of hospitalized COVID-19 patients in Chile

**DOI:** 10.3389/fmed.2023.1164615

**Published:** 2023-08-28

**Authors:** Matías Sanhueza, Manuel Barrera, Juan C. Pedemonte, Luis Rojas

**Affiliations:** ^1^School of Medicine, Department of Internal Medicine, Pontificia Universidad Católica de Chile, Santiago, Chile; ^2^School of Medicine, Division of Anesthesiology, Pontificia Universidad Católica de Chile, Santiago, Chile; ^3^School of Medicine, Program of Pharmacology and Toxicology, Pontificia Universidad Católica de Chile, Santiago, Chile

**Keywords:** CALL score, COVID-19, mortality, prediction, inpatients

## Abstract

**Introduction:**

The CALL score is a predictive tool for respiratory failure progression in COVID-19. Whether the CALL score is useful to predict short- and medium-term mortality in an unvaccinated population is unknown.

**Materials and methods:**

This is a prospective cohort study in unvaccinated inpatients with a COVID-19 pneumonia diagnosis upon hospital admission. Patients were followed up for mortality at 28 days, 3, 6, and 12 months. Associations between CALL score and mortality were analyzed using logistic regression. The prediction performance was evaluated using the area under a receiver operating characteristic curve (AUROC).

**Results:**

A total of 592 patients were included. On average, the CALL score was 9.25 (±2). Higher CALL scores were associated with increased mortality at 28 days [univariate: odds ratio (OR) 1.58 (95% CI, 1.34–1.88), *p* < 0.001; multivariate: OR 1.54 (95% CI, 1.26–1.87), *p* < 0.001] and 12 months [univariate OR 1.63 (95% CI, 1.38–1.93), *p* < 0.001; multivariate OR 1.63 (95% CI, 1.35–1.97), *p* < 0.001]. The prediction performance was good for both univariate [AUROC 0.739 (0.687–0.791) at 28 days and 0.869 (0.828–0.91) at 12 months] and multivariate models [AUROC 0.752 (0.704–0.8) at 28 days and 0.862 (0.82–0.905) at 12 months].

**Conclusion:**

The CALL score exhibits a good predictive capacity for short- and medium-term mortality in an unvaccinated population.

## Introduction

In December 2019, a new strain of coronavirus (SARS-CoV-2) was identified in Wuhan, China, leading to an unprecedented pandemic (1). On 3 March 2020, the first case was reported in Chile. Since then, the number of hospitalizations for pneumonia associated with acute respiratory failure has increased dramatically, putting a significant strain on both the public and private healthcare systems (2).

The coronavirus disease (COVID-19) mainly affects the respiratory system and has also been associated with coagulopathy and disturbances in neurological, cardiac, hepatic, and renal functions (1). A study conducted in the United Kingdom reported that 17.1% of patients hospitalized with COVID-19 were admitted to an intensive care unit (ICU), and 55% of all patients required high-flow oxygen therapy at some point during their hospitalization (3). In-hospital mortality was estimated at around 15–20% (1). These data were published before the availability of steroids, immunomodulators, and vaccination (9–11).

The response to the COVID-19 pandemic required a systematic and collaborative approach. From the moment the first cases of SARS-CoV-2 virus infection were detected worldwide, the scientific community came together to develop an effective strategy to fight the pandemic. Several vaccines were developed and underwent rigorous clinical trials to assess their safety and efficacy. The trials showed that the vaccines were 95% or more effective in preventing severe COVID-19 infections and ~50% effective in preventing mild infections (14). The vaccination has significantly increased the immunity rate among the population, leading to a decrease in COVID-19-related infections and hospitalizations (14). However, there is still a small percentage of the population that remains unvaccinated and may be susceptible to developing severe forms of the disease.

Considering the high mortality observed in unvaccinated patients with COVID-19 pneumonia, the high demand for hospital resources, and the scarcity of effective therapeutic interventions, it is of the utmost concern to create tools that allow for predicting the progression of the disease and mortality risk. Subsequently, this may help in the efficient use of health resources in high-risk patients.

For this purpose, Ji et al. analyzed a retrospective cohort of COVID-19-unvaccinated hospitalized patients (4). They identified risk factors associated with progression to severe respiratory failure, such as the number of comorbidities, advanced age, lymphopenia, and elevated lactate dehydrogenase (LDH) levels. Based on their results, they proposed the Comorbidity, Age, Lymphocyte count, and LDH (CALL) score, with a numerical scale from 4 to 13, that allows the stratification of patients according to the risk of progression (4). In May 2020, Grifoni et al. conducted a preliminary analysis of the score in an Italian cohort of COVID-19 hospitalized patients. They found that the score was useful to predict in-hospital mortality at 28 days from admission, perhaps even superior to its ability to establish the risk of progression to severe respiratory failure (5). Other groups have proposed other scores with similar purposes, including clinical, laboratory, and imaging variables. Although these scores comprise more variables, some are expensive (computed tomography and interpretation by expert radiologists), include exams that are not always available (myoglobin), and have not been internally and/or externally validated to our knowledge (6–8). The CALL score only requires clinical variables and laboratory tests that are inexpensive and widely available. It is simple and easy to use; however, its ability to predict mortality in unvaccinated individuals is still uncertain.

This study aimed to determine the ability of the CALL score to predict increased mortality risk in the unvaccinated Chilean population with a hospital admission diagnosis of COVID-19 pneumonia. The results of this study could validate an easy-to-use prognostic tool and optimize the utilization of hospital resources, therapeutic interventions, and outpatient follow-up.

## Materials and methods

An observational, prospective study was performed in a non-concurrent cohort. The execution of this project was approved by the Research Ethics Committee of the School of Medicine of the Pontificia Universidad Católica de Chile (ID: 210314003). All patients hospitalized in low- and medium-complexity units of the Hospital Clinico UC CHRISTUS were enrolled in Santiago, Chile, from 18 May 2020 to 31 July 2020. The inclusion criteria were patients aged 18 years or older, with a diagnosis of COVID-19 confirmed by polymerase chain reaction, and who had the necessary variables to calculate the CALL score at the time of admission. The exclusion criteria were patients with end-of-life care established at hospital admission, a pulmonary infection caused by a different microorganism, and pregnant women. The mortality follow-up was conducted at 28 days, 3 months, 6 months, and 12 months after hospital admission. Mortality was defined as any cause of death, related or not to the respiratory condition, during and after hospitalization.

### Data collection

Clinical and laboratory data were obtained from the medical and nursing records and the hospital's electronic system: demographic data (sex, age, and date of birth), date and unit of admission, comorbidities [such as chronic kidney disease, diabetes mellitus type 1 or 2, hypertension, heart failure, coronary artery disease or previous stroke, atrial fibrillation, chronic obstructive pulmonary disease (COPD)/asthma or other respiratory disease, obesity, cirrhosis, thromboembolic disease, solid or hematologic malignancy, and history of transplantation or immunosuppression], and laboratory tests included in the CALL score (absolute lymphocyte count and LDH). Mortality was obtained from death certificates issued by the Chilean Civil Registry Service. Finally, other clinical variables were observed: respiratory support on admission, progression to severe respiratory insufficiency [defined as PaFi < 150 or need of high-flow nasal cannula (HFNC), non-invasive ventilation (NIV) and/or intermittent mandatory ventilation (IMV)], suspected bacterial superinfection, thromboembolic disease, suspected organizing pneumonia (OP), and use of corticosteroids and/or tocilizumab.

### Sample size

Assuming a “rule of thumb” of 10 events for each dimension (covariate) included in a logistic regression model to predict mortality (dichotomous variable), a sample size of 475 patients was estimated, which would be adequate for modeling. In addition, a sample loss of 20% was considered. Thus, 570 patients were considered sufficient to perform the analysis.

### Statistical analysis

For data analysis, patients were grouped according to their CALL score: mild (4–6 points), moderate (7–9 points), and severe (10–13 points). Continuous variables are presented as medians and interquartile ranges, and categorical variables are presented as numbers and percentages. Comparisons were made by ANOVA or the Kruskal–Wallis test and the chi-squared test or Fisher's test, respectively, depending on the distribution. Predictive analysis between the CALL score and different mortality time points (28 days and 12 months) was explored with logistic regression in univariate and multivariate analyses. Covariates of events during hospitalization {progression of respiratory failure, thromboembolic event [venous thromboembolism (VTE)], bacterial superinfection, OP, and use of corticosteroids and/or tocilizumab} were included as confounding variables. The interaction between these variables was not explored. Prediction performance was assessed by calculating the area under a receiver operating characteristic (ROC) curve. Sensitivity and specificity values were calculated for the best score performance in the univariate analysis. A *p*-value of < 0.05 was considered statistically significant. The analyses were performed in R software.

## Results

A total of 592 patients were enrolled, of which 56% were men. The mean age was 60.82 ± 15.42 years. Overall, 68% of patients were admitted on room air, 57% had a CURB-65 score of < 2, and 60% were hospitalized in a low-complexity unit (floor). The average length of stay (LOS) was 8 (range 4–15) days, with the following events occurring during hospitalization: 39% progressed to severe respiratory insufficiency, 27% were assumed to be affected by bacterial superinfection, and 8% were diagnosed with VTE. Regarding treatment, 66% received systemic corticosteroids, and 8.6% received intravenous tocilizumab. All patients were unvaccinated. Overall mortality at 28 days was 8.8%, at 3 months was 9.5%, at 6 months was 9.6%, and at 1 year was 10% (Table 1).

**Table 1:** Characteristics and mortality of COVID-19 inpatients.

		**Call score**			
		**Low risk**	**Intermediate risk**	**High risk**	
***n***	**592**	**83**	**198**	**311**	***p***
Age	60.82 ± 15.42	44.84 ± 10.21	55.33 ± 14.46	68.57 ± 12.05	< 0.001
Male (%)	56.1	54.2	57.6	55.6	0.851
Smokers (%)	20.9	22.9	15.1	24.2	< 0.001
No of comorbidities	1 (0–2)	0	0 (0–1)	1 (1,2)	< 0.001
Admission respiratory rate	28.45 ± 5.21	28.09 ± 4.93	28.35 ± 5.38	28.62 ± 5.18	0.676
Respiratory support in the ED (%)					< 0.001
Room air	68.2	83.1	77.3	58.2	
Nasal cannula	5.9	6	6.1	5.8	
Venturi mask/non-rebreather mask	23.5	9.6	14.6	32.8	
HFNC/NIPPV	2	1.2	1.5	2.6	
**Laboratory in the ED**					
BUN (mg/dL)	14 (11–20)	14.5 (11–20)	13 (10–19)	14 (11–20.25)	0.498
Admission unit (%)					< 0.001
Floor	60.3	67.5	72.2	50.8	
Intermediate care unit	31.2	24.1	18.7	41.2	
ICU	8.4	8.4	9.1	8	
CURB-65 (%)					< 0.001
< 2	56.9	74.7	66.7	46	
≥2	32.1	12	18.1	47	
LOS (days) (x)	8 (4–15)	5 (3–8)	7 (4–12)	10.5 (6–21)	< 0.001
Bacterial coinfection (%)	5.9	0	4	8.7	0.005
Bacterial sepsis during stay (%)	27	13.2	18.1	36.3	< 0.001
VTE (%)	7.8	8.4	5.1	9.3	0.208
COP (%)	14.9	7.2	13.6	17.7	0.137
LTE (%)	7.1	0	3	11.6	< 0.001
Severe respiratory insufficiency (%)	38.9	22.9	27.8	50.2	< 0.001
Steroids (%)	65.9	41	60.6	75.9	< 0.001
Tocilizumab (%)	8.6	7.2	9.1	8.7	0.878
**Mortality**					
28 day (%)	8.8	0	3.5	14.5	< 0.001
3 months (%)	9.5	0	3.5	15.8	< 0.001
6 months (%)	9.6	0	3.5	16.1	< 0.001
1 year (%)	10	0	3.5	16.1	< 0.001

The mean CALL score was 9.25 (±2) points. Mortality was significantly higher in the severe group (15.8% at 3 months and 16.1% at 1 year; *p* < 0.001). All patients in the mild group were alive during the 1-year follow-up (Table 1).

Univariate and multivariate logistic regression were performed for 28-day and 1-year mortality, adjusted for the following confounders: ventilatory support on admission, VTE during hospitalization, bacterial superinfection, OP, corticosteroid use, and tocilizumab use. For 28-day mortality, univariate analysis revealed an odds ratio (OR) of 1.58 (95% CI, 1.34–1.88), with a *p*-value of < 0.001 and multivariate analysis revealed an OR of 1.54 (95% CI, 1.26–1.87), with a *p*-value of < 0.001. For 1-year mortality, univariate analysis revealed an OR of 1.63 (95% CI, 1.38–1.93), with a *p*-value of < 0.001 and multivariate analysis revealed an OR of 1.63 (95% CI, 1.35–1.97), with a *p*-value of < 0.001.

**Figure 1:**
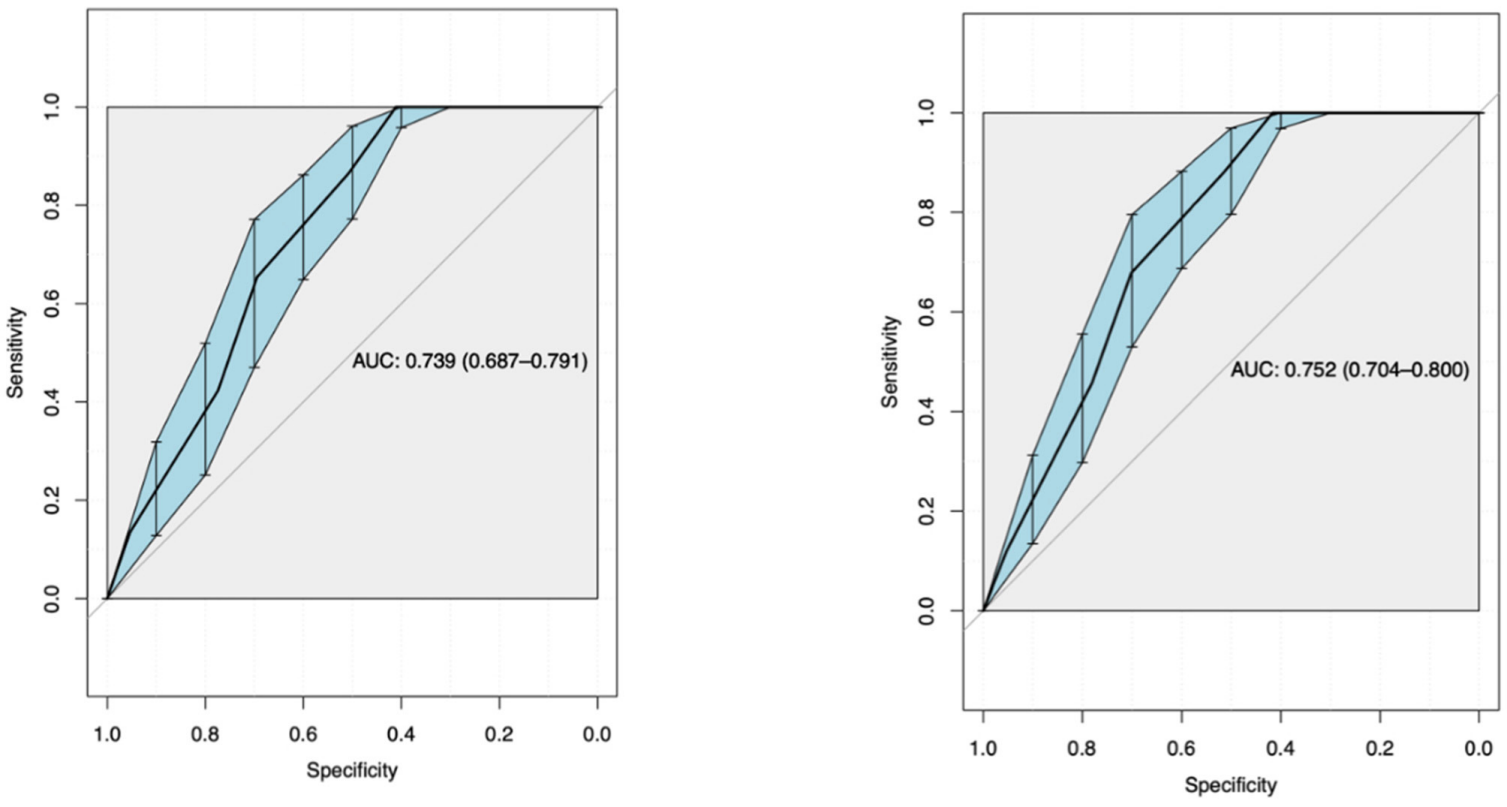
Univariate analysis ROC curve.

**Figure 2:**
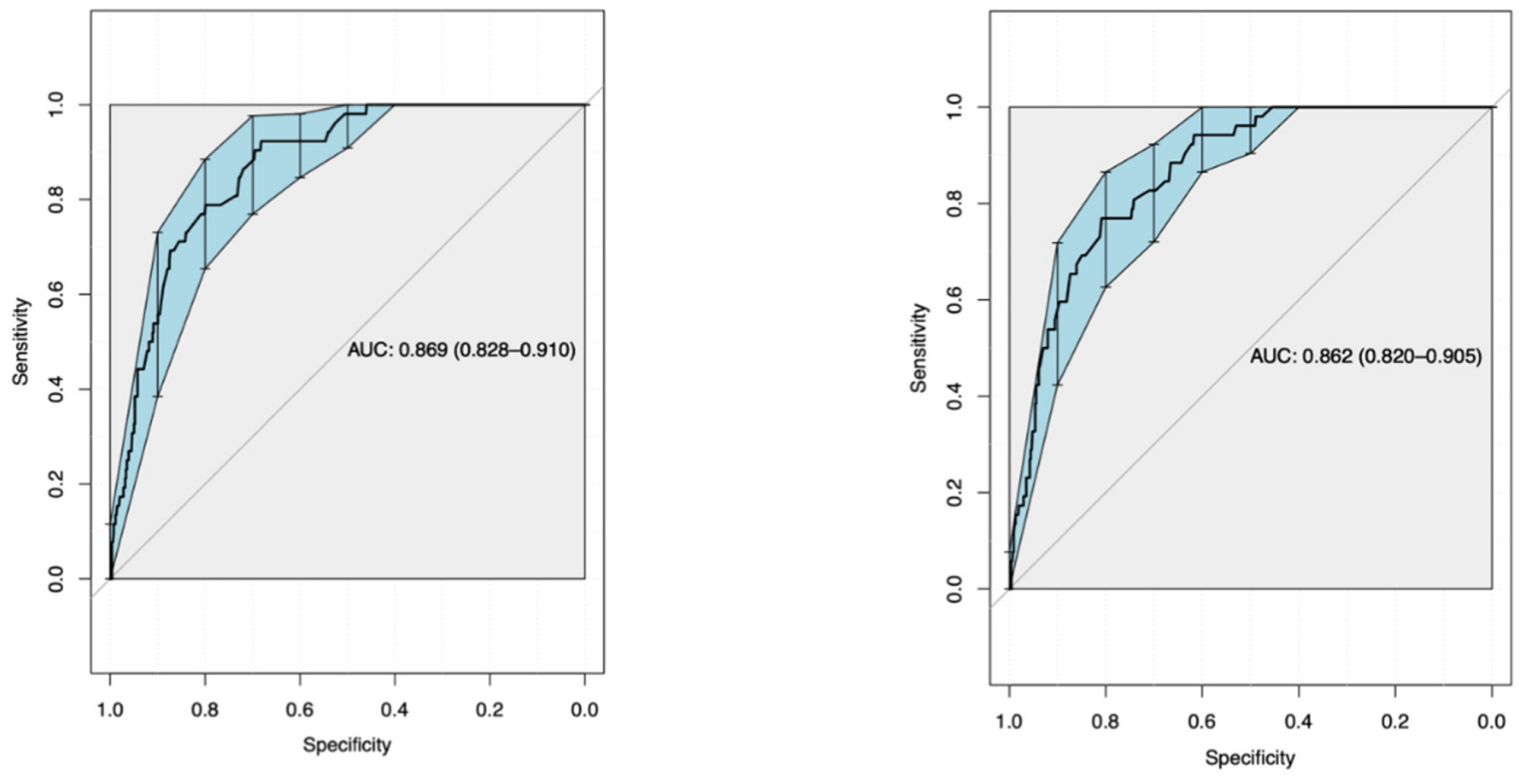
Multivariate analysis ROC curve.

Using ROC curve analysis, a univariate AUROC of 0.739 (0.687–0.791) and a multivariate AUROC of 0.752 (0.704–0.8) were obtained for 28-day mortality. Univariate AUROC for 1-year mortality was 0.869 (0.828–0.91) and multivariate AUROC was 0.862 (0.82–0.905) (Figures 1, 2). It was determined that, for all univariate models, the cutoff score (threshold) of the CALL score was equal to 8.5, with a sensitivity of 100% and a specificity of 40%.

## Discussion

The severe acute respiratory syndrome coronavirus 2 (SARS-CoV-2) infection has proven to be a highly morbid and lethal disease worldwide. Given the high demand for hospital care, many efforts have been focused on developing effective ways to predict which patients are at higher risk of severe disease and mortality. This may enable the efficient use of medical resources and increase the therapeutic effect. The CALL score emerged as a method with good accuracy and easy-to-use features, and it achieved optimal prediction of progression. Nevertheless, mortality risk prediction remained unknown. This study evaluated the CALL score for mortality risk prediction, suggesting that it effectively predicts short- and long-term mortality in an unvaccinated Chilean population.

Multivariate analysis revealed the strong predictive power of the CALL score, with each point increase correlated with a 54% increase in 28-day mortality risk. Although the unadjusted analysis showed a sensitivity of 100%, it came at the expense of relatively low specificity (40%), which is a typical tradeoff in diagnostic performance search tools. Another surprising finding from this analysis was that most of the mortality occurred mainly within the first 3 months and was negligible when the score was classified as low risk. This information has profound implications as it guides clinicians in making crucial decisions in emergency departments, during hospitalization, and for outpatient follow-up, leading to more efficient use of resources and targeted interventions.

Previously, only Grifoni et al. tested the CALL score as a predictor of hospital mortality. They reported good predictive power [AUC, 0.768 (95% CI, 0.705–0.823)]. However, they did not report confounding analyses or drug co-interventions, and the sample size was small (only 210 patients).

Despite the wide availability of vaccines and their significant impact in reducing mortality related to COVID-19, there is still a considerable percentage of the population that remains unvaccinated, either due to unequal access to the vaccine, rejection of vaccination, or medical contraindications. The continued relevance of the CALL score for these populations cannot be underestimated. Even among vaccinated patients, immunosuppressed patients have been known to develop a weaker immune response, making the CALL score a potentially valuable tool for risk stratification within this subgroup.

The CALL score, with its simplicity and reliance on widely available clinical and laboratory parameters, presents a universally applicable tool that is especially useful in resource-limited settings. As such, its predictive power should ideally be reassessed in the current context, with new variables such as vaccination status and new treatments included.

This study has some strengths, including the prospective design, sufficient sample size, statistical power to explore the association between the CALL score and mortality, and the use of multivariate analysis to control for confounding factors. However, some limitations need to be addressed. First, the study was conducted with patients from only one health center. Second, the results are limited to unvaccinated patients, which restricts the generalization of the conclusions. Third, the research for this study was conducted in the early phases of the pandemic when current therapies were not available. Finally, another important limitation is that the BMI index, which is a high-risk factor associated with the progression or severity of COVID-19, was not reported (12, 13).

In conclusion, the results of this study suggest that the CALL score achieved an optimal prediction of both short- and long-term mortality in this population. This easy-to-use score makes it a universally applicable and potentially valuable tool in managing unvaccinated COVID-19 inpatients, especially in resource-limited settings. The score could guide the implementation of more aggressive intervention strategies, closer monitoring, and the allocation of additional resources for patients with high CALL scores.

## Data availability statement

The original contributions presented in the study are included in the article/supplementary material, further inquiries can be directed to the corresponding author.

## Ethics statement

The execution of this project was approved by the Research Ethics Committee of the School of Medicine of the Pontificia Universidad Católica de Chile (ID: 210314003). The studies were conducted in accordance with the local legislation and institutional requirements. The Ethics Committee/Institutional Review Board waived the requirement of written informed consent for participation from the participants or the participants' legal guardians/next of kin because no use of sensitive data, pandemic restrictions.

## Author contributions

MS and MB were responsible for designing the project, gathering the data, writing the manuscript, and performing the data analysis. JP was responsible for choosing the statistical analysis and developing it, including the creation of ROC curves. LR provided guidance and made critical corrections. All authors contributed to the article and approved the submitted version.
